# Nasal Herpes Simplex With Infraorbital Neuralgia: A Rare Presentation

**DOI:** 10.7759/cureus.49584

**Published:** 2023-11-28

**Authors:** Smriti Wadhwa, Shraddha Jain, Rinkle Gemnani, Bhushan Madke

**Affiliations:** 1 Department of Otorhinolaryngology, Jawaharlal Nehru Medical College, Datta Meghe Institute of Higher Education and Research, Wardha, IND; 2 Department of Medicine, Jawaharlal Nehru Medical College, Datta Meghe Institute of Higher Education and Research, Wardha, IND; 3 Department of Dermatology, Venereology, and Leprosy, Jawaharlal Nehru Medical College, Datta Meghe Institute of Higher Education and Research, Wardha, IND

**Keywords:** infraorbital nerve, nose, trigeminal neuralgia, neuralgia, herpes simplex

## Abstract

Facial herpes is a form of herpes simplex type I infection and presents with characteristic vesicular lesion around the perioral region. Nasal herpes, a form of facial herpes is a rare presentation with only a few cases reported in the literature. Neuralgic pain in herpes simplex is usually experienced at the site of the lesion during or before the eruptive stage. Here, we are reporting a case where the patient with a herpes simplex lesion over the tip of her nose presented with pain over the infraorbital region, which is a region supplied by the infraorbital nerve, a branch of the maxillary division of the trigeminal nerve. Initially confused as a bacterial infection due to its unusual presentation and rarity of the condition, the patient was given anti-bacterial therapy, but on showing no relief in symptoms, the patient was treated with appropriate antiviral drugs, following which complete remission of the lesion was observed. The case highlights a rare site for a common condition and atypical presentation.

## Introduction

Herpes simplex virus (HSV) is one of the most prevalent human viruses worldwide. Skin and mucosal infection can occur at any anatomic site; however, the most common sites are the oral, labial, and urogenital areas and sometimes the lung and conjunctival areas [1]. It manifests in two forms: HSV type 1 (HSV-1) and HSV type 2 (HSV-2). The most common manifestations of HSV-1 are cold sores and facial herpes, others being stromal keratitis, and occasionally meningitis and encephalitis, in immunocompromised individuals [1,2]. Nasal herpes, a type of facial herpes is caused by HSV infection over the dorsum of the nose or intranasally. Intranasal forms presenting as mass (hypertrophic lesion) over inferior turbinates or mimicking acute invasive fungal sinusitis in immunocompromised individuals have been reported [1,3]. Isolated mild external nasal lesions without involvement of other facial sites are a rare occurrence. External nasal lesions are mostly reported in immunocompromised patients and are of severe type [4]. Neuralgic pain in the distribution of ophthalmic division of the trigeminal nerve is more common in herpes zoster infection [5] and rare in herpes simplex. Here we report a case of herpes simplex infection involving the nasal tip, presenting with isolated vesicular lesions of mild form, and infraorbital neuralgia in the distribution of the infraorbital nerve, a branch of the maxillary division of the trigeminal nerve.

## Case presentation

**Figure 1:**
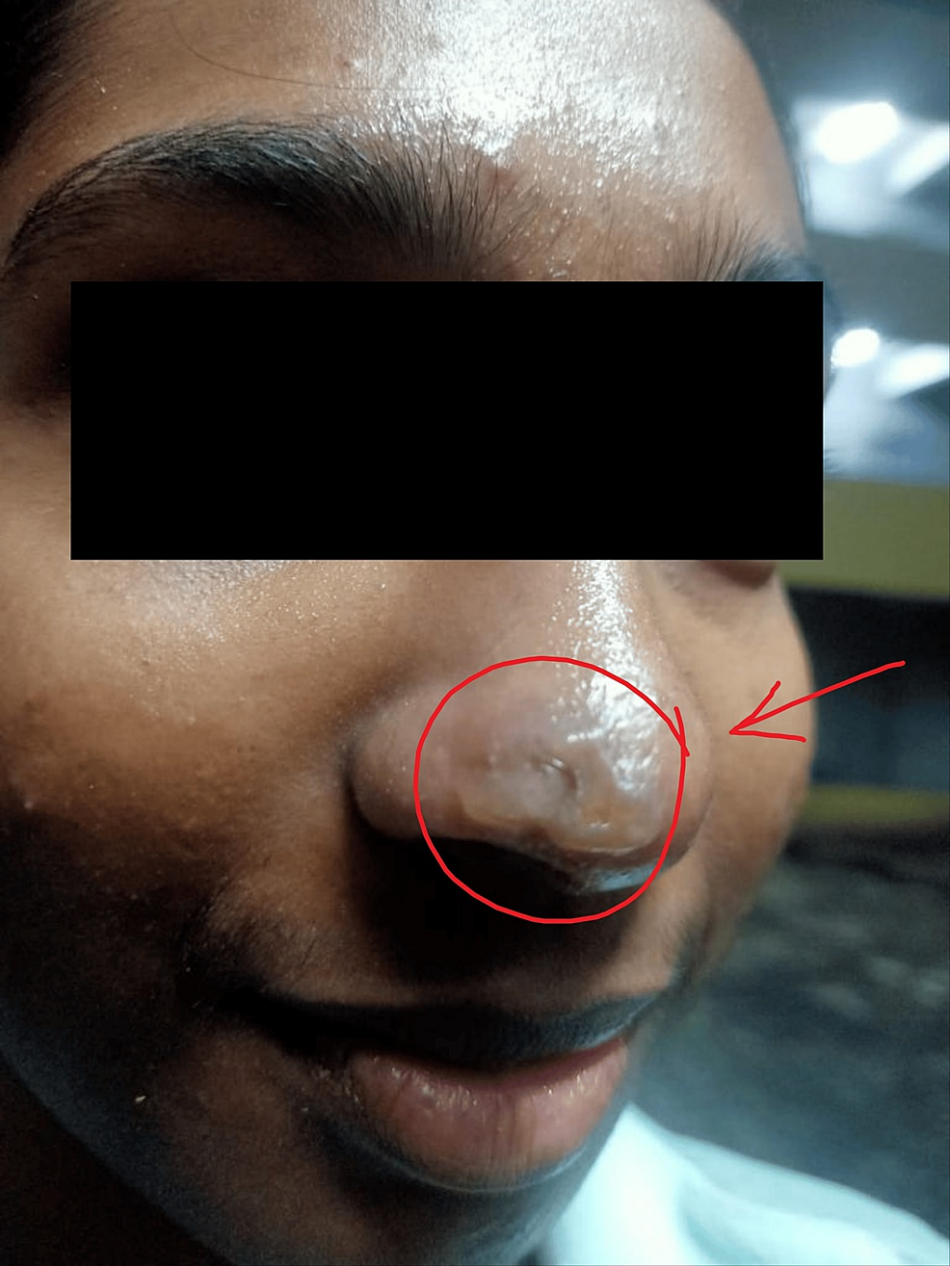
Vesicular blister-like lesion over the nose (within circle indicated with red arrow). Image Credit: Author Smriti Wadhwa

A 22-year-old female medical student sought medical attention due to a troubling blister that had developed on the tip of her nose and extended up to the right ala (Figure 1). This blister was accompanied by intense pain that radiated to various areas, including below her right eye, the base of her nose, cheek, and temple, and was also associated with intermittent generalized headaches. Upon taking her medical history, it was revealed that she had been experiencing a mild fever for the past week, which was attributed to an upper respiratory tract infection (URTI). The URTI also presented symptoms like nasal discharge and itching on the skin of her nose. She had no history of facial herpes in the past. Additionally, her medical records indicated that she had a pre-existing history of migraine and chronic tonsillitis. She had no otologic signs or symptoms and facial nerve examination was found to be normal. The complete blood counts of the patient were within normal limits and so were the kidney function tests, liver function tests, and fasting and post-prandial blood sugar among the other hematological investigations.

The patient initially received intravenous antibacterial therapy in the form of Injectable amoxicillin-clavulanic acid 1.2gm 12 hourly, which was continued for two days, and topical application of mupirocin ointment, in the emergency ward, considering the possibility of nasal furunculosis with vestibulitis due to rarity of this site for herpes simplex. However, the patient's condition did not show any improvement, and the diagnosis of herpes simplex type 1 was made primarily on the basis of clinical suspicion. Additionally, on serology, the patient was found to be IgM seropositive for HSV-1.

The patient then received valacyclovir, an antiviral medication, in an oral dosage of 1000mg twice daily for a period of seven days. This treatment resulted in a reduction of blister size and the formation of crusts. Upon completing the full course of valacyclovir, the patient achieved complete remission of the blister, without any scarring, signifying the successful resolution of the herpes simplex infection.

## Discussion

The incidence of nasal herpes is not very common worldwide. Cutaneous blister-like lesions over the nose can be caused by infective conditions like erysipelas, impetigo, vestibulitis caused by staphylococcal infection, chicken pox, herpes zoster, herpes simplex, hand, foot, and mouth disease, syphilis, and mycoplasma infection [6], and also autoimmune causes like dermatitis herpetiformis or acute allergic contact dermatitis [7]. In the present case, the patient presented with a vesicular blister-like lesion over the tip of her nose, so other more common differentials like vestibulitis were initially considered.

However, the severity of pain was out of proportion to the swelling and there was no edema over the face or periorbital region. The pain in the infraorbital region and the dorsum of her nose was hence thought of as neuralgia, and a diagnosis of herpes simplex was made based on clinical suspicion and viral serology. Pain in herpes simplex usually corresponds to the area around the vesicles and could be dull aching pain or sharp shooting pain, or there could be a burning or itching sensation, prickling paresthesias, or dysesthesias [8]. It usually occurs before or during the eruptive stage. Sensation of the nose comes from the ophthalmic (V1) and maxillary (V2) divisions of the trigeminal nerve. The maxillary division (V2) provides bilateral innervation to the lateral dorsum and alae [9]. Since lesions were on the ala on the right side, the patient experienced pain in the distribution of the infraorbital nerve, a branch of maxillary (V2) divisions of the trigeminal nerve. It is rare to have neuralgic pain in herpes simplex; it is more common in herpes zoster infection [5].

Most people get infected with HSV-1 before they turn 30 years old. HSV infections are contracted sub-clinically, mostly in childhood via mucosa or rubbed skin through saliva or contact, sharing cosmetics/drinkware, or among medical professionals, [10] entering dermal and epidermal cells. Initial exposure can be asymptomatic or manifest as herpetic gingivostomatitis usually with systemic signs and symptoms like fever, headache, malaise, myalgia, lymphadenopathy, localized pain, and pruritus. It starts with macules and papules, then blisters are formed (a hallmark of HSV-1). The time from exposure to symptom varies from one to 26 days. Blisters form pustules, bursting after two days, causing ulcer and scab formation in 96 hours [11]. In our patient, this episode appeared to be the first episode and was preceded by fever.

The virus replicates in the ganglia and travels through the olfactory or trigeminal tracts. Herpes simplex can rarely accompany facial nerve palsy. HSV-1 becomes latent in the sensory neurons and can reactivate due to factors like cold, fever, stress, ultraviolet exposure, menstruation, orofacial trauma/surgery, and immunosuppression [12,13].

Diagnosis is done by staining, culture, serological tests, and polymerase chain reaction [1]. However, the role of these tests in diagnosis with regard to their sensitivity and specificity is controversial and also depends on the method and kit used for testing [1,14]. Hence, clinical judgment is crucial and can be aided by laboratory tests.

Acyclovir 400mg orally three times daily or 200mg five times daily or valacyclovir 1g twice daily for seven to 10 days with topical administration of analgesics like lidocaine or benzocaine and/or topical acyclovir ointment is the treatment advocated for facial herpes simplex infection. Oral therapy is thought to be more effective than topical therapy. Oral medications for patients with recurrence include acyclovir (400mg three times a day for five days) and valacyclovir (2g twice daily for one day) [15]. There is a role of topical steroids in the treatment of UV-induced herpes simplex [2,16,17]. Since the lesion and neuritis responded promptly to antivirals alone, steroids were not given, and also, it was the first episode with no history of sun exposure.

## Conclusions

Nasal herpes simplex is a rare differential diagnosis for eruptive lesions over the nose. It can present with neuralgic symptoms in the form of infraorbital neuralgia, a rare manifestation of herpes simplex. A high index of suspicion and prompt treatment with antivirals can result in complete remission without any complications. This case aims to describe a common condition of facial herpes simplex at an unusual site, i.e., the tip of the nose, as isolated lesions and presenting with rare characteristics and distribution of pain, in order to differentiate it from herpes zoster and other vesicular lesions over the tip of the nose.
